# Eosinophil-to-Neutrophil Ratio Predicts Poor Prognosis of Acute Ischemic Stroke Patients Treated With Intravenous Thrombolysis

**DOI:** 10.3389/fneur.2021.665827

**Published:** 2021-07-12

**Authors:** Haoye Cai, Honghao Huang, Chenguang Yang, Junli Ren, Jianing Wang, Beibei Gao, Wenjing Pan, Fangyue Sun, Xinbo Zhou, Tian Zeng, Jingyu Hu, Yilin Chen, Shunkai Zhang, Guangyong Chen

**Affiliations:** ^1^Department of Rehabilitation Medicine, The Third Affiliated Hospital of Wenzhou Medical University, Wenzhou, China; ^2^Department of Neurology, The Third Affiliated Hospital of Wenzhou Medical University, Wenzhou, China; ^3^School of the First Clinical Medical Sciences, Wenzhou Medical University, Wenzhou, China; ^4^Department of Internal Medicine, The Third Affiliated Hospital of Wenzhou Medical University, Wenzhou, China

**Keywords:** ischemic stroke, inflammation, prognosis, thrombolysis, eosinophil-to-neutrophil ratio

## Abstract

**Background and Purpose:** The eosinophil-to-neutrophil ratio (ENR) was recently reported as a novel inflammatory marker in acute ischemic stroke (AIS). However, few studies reported the predictive value of ENR in AIS patients, especially for those with intravenous thrombolysis.

**Methods:** Two hundred sixty-six AIS patients receiving intravenous thrombolysis were retrospectively recruited in this study and followed up for 3 months and 1 year. The Modified Rankin Scale (mRS) and the time of death were recorded. Poor outcome was defined as mRS 3–6. After excluding patients who were lost to follow-up, the remaining 250 patients were included in the 3-month prognosis analysis and the remaining 223 patients were included in the 1-year prognosis analysis.

**Results:** ENR levels in the patients were lower than those in the healthy controls. The optimal cutoff values for the ability of ENR × 10^2^ to predict 3-month poor outcome were 0.74 with 67.8% sensitivity and 77.3% specificity. Patients with ENR × 10^2^ ≥ 0.74 have a lower baseline National Institutes of Health Stroke Scale (NIHSS) score (median: 7 vs. 11, *p* < 0.001). After multivariate adjustment, patients with ENR × 10^2^ ≥ 0.74 were more likely to come to a better 3-month outcome (OR = 0.163; 95% CI, 0.076–0.348, *p* < 0.001). At the 1-year follow-up, the patients with ENR × 10^2^ ≥ 0.74 showed a lower risk of mortality (HR = 0.314; 95% CI, 0.135–0.731; *p* = 0.007).

**Conclusions:** A lower ENR is independently associated with a 3-month poor outcome and a 3-month and 1-year mortality in AIS patients treated with intravenous thrombolysis.

## Introduction

Stroke is one of the leading causes of mortality and morbidity worldwide (1). Intravenous thrombolysis with recombinant tissue plasminogen activator (r-tPA) was recommended for acute ischemic stroke (AIS) patients within 4.5 h of stroke onset, and an increasing trend of r-tPA treatment was discovered over the past 13 years (2). However, there were still nearly half of patients who went into major disability or died after 3 months of stroke onset. Hence, it was vital to find an accurate and concise prognostic marker to better distinguish patients who have a higher risk for poor outcome.

A strong neuro-inflammatory response is characteristic of ischemic stroke (3). Neutrophil plays an important role in the vascular innate immune system, and its distribution was highly influenced by the administration r-tPA (4). A higher neutrophil level after r-tPA infusion is a predictive factor for parenchymal hemorrhage and poor function outcome of AIS (5). Another notable aspect of the acute inflammatory response involves a sustained and rapid reduction of blood eosinophil count (6). A previous study reported that eosinopenia is associated with severe stroke and poor prognosis the day after admission (7). In addition, without concomitant eosinopenia, high neutrophil counts alone may not predict for a short-term risk of mortality of AIS patients (8), suggesting a potential interaction between eosinophils and neutrophils in ischemic stroke.

The eosinophil-to-neutrophil ratio (ENR) is a novel biomarker that was reported to be associated with in-hospital mortality of patients with chronic obstructive pulmonary disease (COPD) (9). A recent study reported that a neutrophil-to-eosinophil ratio represents systemic inflammation and a higher neutrophil-to-eosinophil ratio at admission is related to higher odds of in-hospital mortality in AIS patients (10). However, limited by the accuracy of the instrument, eosinophil count may show a number of 0 in some patients and excluding these patients could introduce some bias. Therefore, ENR may be a more stable biomarker than the neutrophil-to-eosinophil ratio. We performed this retrospective observational cohort study, aiming to analyze the predictive value of ENR for the 3-month and 1-year prognosis of AIS patients treated with r-tPA intravenous thrombolysis.

## Materials and Methods

### Data Availability

The data that support the findings of this study are available from the corresponding author on reasonable request.

### Study Population

**Figure 1:**
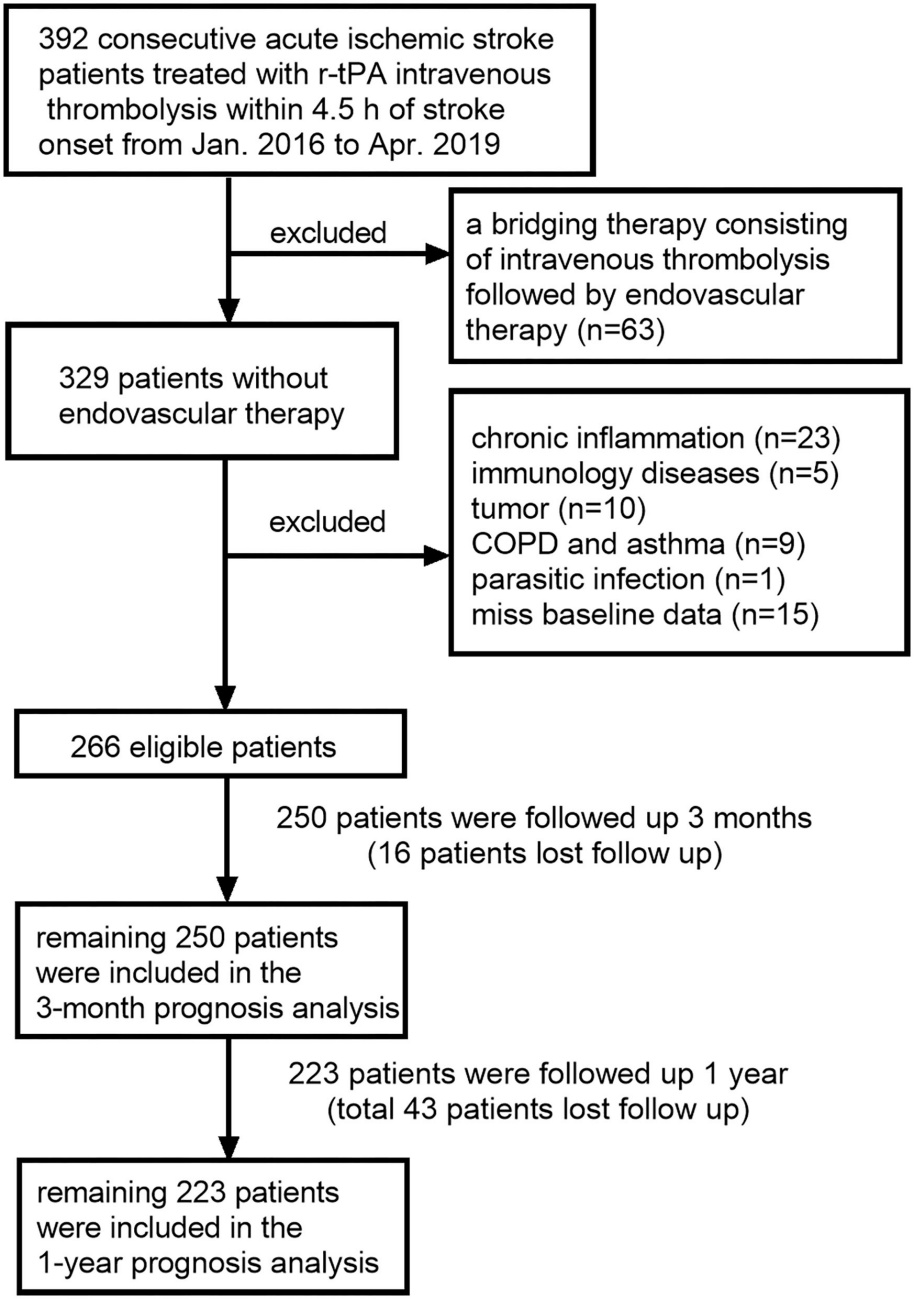
Flowchart for patient selection.

The detailed selection criteria of the study patients are displayed in Figure 1. A total of 266 AIS patients who were treated with intravenous r-tPA (0.9 mg/kg body weight, maximum 90 mg, 10% of the dose as a bolus, followed by a 60-min infusion) from January 2016 to April 2019 at the Third Affiliated Hospital of Wenzhou Medical University and 2,196 healthy controls (HCs) were evaluated in this retrospective study. Patients were excluded if they have (1) a bridging therapy; (2) chronic inflammation; (3) immunology diseases; (4) tumor; (5) COPD or asthma; (6) parasitic infection, and (7) no full baseline data. We followed up each patient 3 months and 1 year after AIS onset. After excluding patients lost to follow-up, the remaining 250 patients were included in the 3-month prognosis analysis and the remaining 223 patients were included in the 1-year prognosis analysis. This study was approved by the Ethics Committee of the Third Affiliated Hospital of Wenzhou Medical University and was carried out in accordance with the Declaration of Helsinki.

### Data Collection

Information of HCs was obtained from electronic examination reports. As for patients, the demographic data (age, sex) and medical history (smoking, hypertension, diabetes hyperlipidemia, atrial fibrillation, and prior stroke) were obtained from medical records. National Institutes of Health Stroke Scale (NIHSS) scores on admission and stroke subtypes were evaluated by experienced clinicians. Blood samples were collected on 24 h of admission. ENR was calculated using eosinophil counts divided by neutrophil counts. At 3 months and 1 year after onset of AIS, the prognoses of patients were assessed through telephone follow-up by two clinicians.

### Diagnostic criteria

The etiology of AIS was classified on the basis of the Trial of Org 10,172 in Acute Stroke Treatment (TOAST) criteria: cardioembolic, atherosclerotic, small vessel or lacunar, and cryptogenic or others (11). Stroke severity was assessed using the NIHSS score. A good function outcome was defined as mRS scores of 0–2 while a poor function outcome was defined as mRS scores of 3–6. Outcomes included poor functional outcome and all-cause mortality. A 3-month poor function outcome was regarded as the primary outcome.

### Statistical Analysis

Statistical analyses were performed *via* SPSS Statistics 24.0 software (SPSS Inc., Chicago, IL, USA), R version 4.0.2 (R Foundation for Statistical Computing, Vienna, Austria), and MedCalc Statistical Software version 15.2.2 (MedCalc Software bvba, Ostend, Belgium; http://www.medcalc.org; 2015). Continuous variables were expressed as medians and interquartile range while categorical variables were expressed as frequencies and percentage. The intergroup difference of continuous variables was compared using the Mann–Whitney *U*-test. The chi-square test was performed for categorical variables. ENR levels between HCs and AIS patients were compared before and after age and sex matching. In AIS patients, the optimal cutoff value of ENR to predict the 3-month poor outcome was determined using receiver operating characteristic (ROC) curve analyses, and then patients were divided into a high-ENR group (ENR ×10^2^ ≥ 0.74) and a low-ENR group (ENR × 10^2^ < 0.74). Univariate and multivariable logistic analyses were performed to estimate the association between ENR and AIS outcomes where variables with a *p* < 0.10 in univariate analysis were entered in the multivariable model. In addition, restricted cubic splines with four knots (at the 5th, 35th, 65th, and 95th percentiles) were performed to further investigate the relationship between ENR and AIS outcomes. C-statistics, net reclassification index (NRI), and integrated discrimination improvement (IDI) were employed to assess the incremental predictive ability of ENR. For clinical practice, 1-year mortality was presented graphically using Kaplan–Meier curves and we used log-rank tests to compare survival between high-ENR group and low-ENR group. Cox regression was used to determine whether ENR is a significant predictor for 1-year mortality. Statistical significance was set at *p* < 0.05.

## Results

### Baseline Characteristics of the Study Subjects

Among all enrolled subjects, 266 were AIS patients and 2,196 were HCs. The characteristics of the AIS patients and the HCs are displayed in Table 1. AIS patients were older, having a higher proportion of males than HCs. The higher level of neutrophil count and the lower level of eosinophil count led to lower ENR × 10^2^ in AIS patients (1.19 [0.28–2.90] vs. 3.16 [1.91–5.38]; *p* < 0.001) compared to HCs. After matching of age and sex, ENR × 10^2^ in AIS patients was still lower than that in HCs (1.43 [0.32–2.94] vs. 3.43 [2.27–5.81]; *p* < 0.001).

**Table 1:** Demographic and laboratory characteristics of AIS patients and healthy controls.

	**AIS**	**HCs**	***p-*value**
Before matching	*n* = 266	*n* = 2196	
Age (years)	70 (60–79)	37 (30–46)	<0.001
Sex (male, *n*.%)	166 (62.4)	888 (40.4)	<0.001
Neutrophil (× 10^9^/l)	5.30 (3.88–7.03)	3.14 (2.56–3.90)	<0.001
Eosinophil (× 10^9^/l)	0.06 (0.02–0.12)	0.10 (0.06–0.17)	<0.001
ENR × 10^2^	1.19 (0.28–2.90)	3.16 (1.91–5.38)	<0.001
After matching	*n* = 153	*n* = 153	
Age (years)	62 (56–68)	61 (55–68)	0.799
Sex (male, *n*.%)	91 (59.5)	91 (59.5)	1.000
Neutrophil (× 10^9^/l)	5.10 (3.80–6.80)	2.94 (2.46–3.61)	<0.001
Eosinophil (× 10^9^/l)	0.06 (0.02–0.12)	0.11 (0.06–0.18)	<0.001
ENR × 10^2^	1.43 (0.32–2.94)	3.43 (2.27–5.81)	<0.001

*ENR, eosinophil-to-neutrophil ratio*.

### ENR Cutoff Points Distinguishing a 3-Month Poor Outcome

**Figure 2:**
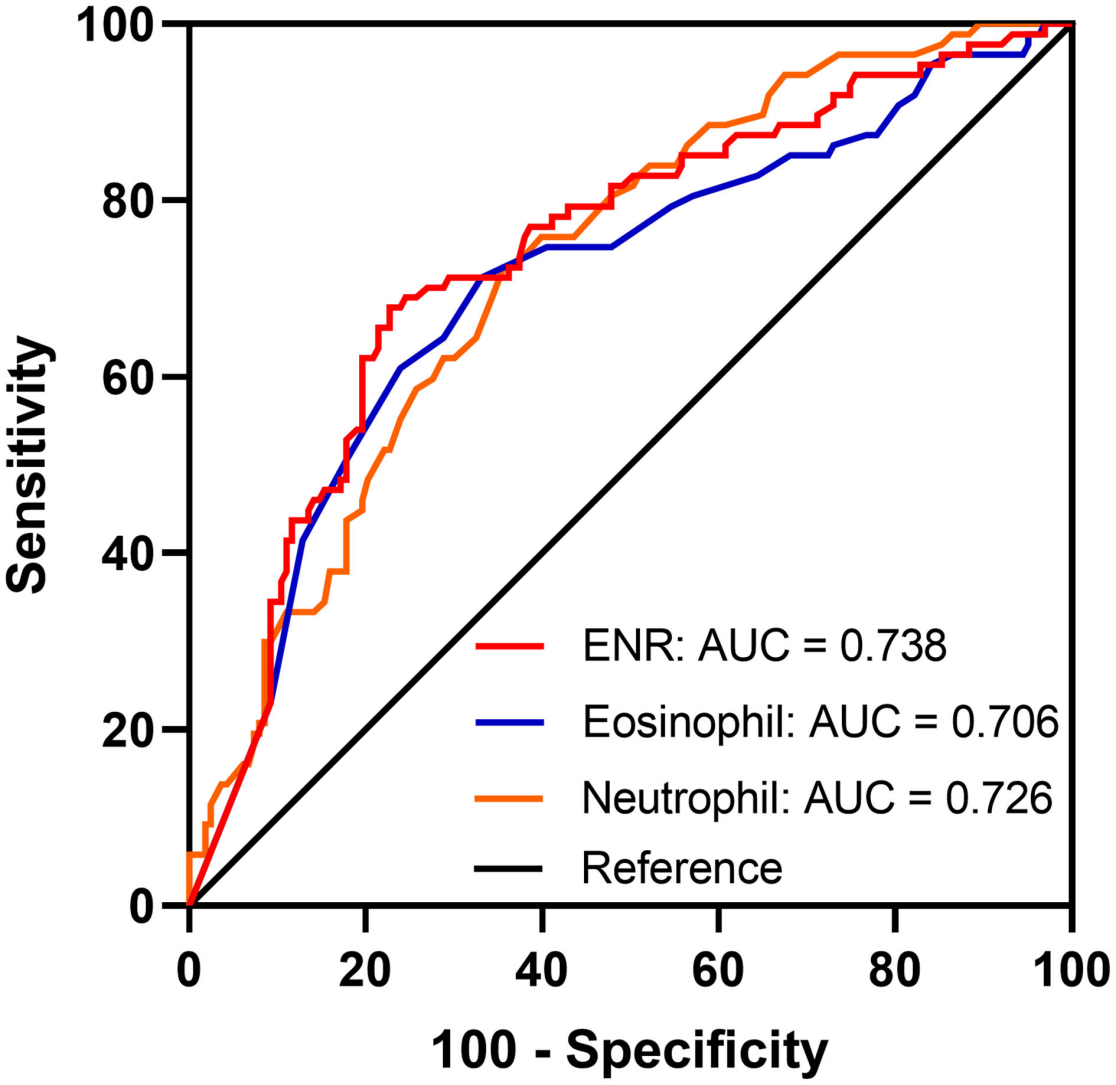
Receiver operator characteristic curves for the prediction of 3-month poor outcome using neutrophil, eosinophil, and eosinophil-to-neutrophil ratio (ENR).

At the 3-month follow-up, 16 (6.0%) patients were lost to follow-up and the remaining 250 patients were included in the prognosis analysis. The optimal cutoff values of the ENR × 10^2^ that best distinguished the 3-month poor outcome were 0.74 with 67.8% sensitivity and 77.3% specificity; the area under the curve (AUC) was 0.738 (95% CI = 0.679–0.792, *p* < 0.001). ENR had a better performance in discriminating patients at high risk and low risk of poor outcome than either eosinophil or neutrophil counts alone (AUC of eosinophil = 0.706; AUC of neutrophil = 0.726) (Figure 2). Patients were divided into a high-ENR group (*n* = 154) and a low-ENR group (*n* = 96) according to the ENR cutoff values. The median ENR × 10^2^ was 2.45 in the high-ENR group and 0.15 in the low-ENR group. A significant higher proportion of male, eosinophil count, and percentage of atherosclerotic stroke and a significant lower percentage of atrial fibrillation, neutrophil count, percentage of cardioembolic stroke, baseline NIHSS score, and 3-month mRS scores were observed in the high-ENR group (Table 2).

**Table 2:** Comparisons of baseline characteristics and 3-month outcomes between ENR groups.

**Variable**	**ENR × 10^**2**^ < 0.74 (*n* = 96)**	**ENR × 10^**2**^ ≥ 0.74 (*n* = 154)**	***p*-value**
**Demographic data**			
Age, (years)	68 (59–80)	70 (60–77)	0.883
Sex, (male, *n*.%)	49 (51.0)	108 (70.1)	0.002
**Stroke risk factors**			
Current smoking, *n* (%)	17 (17.7)	39 (25.3)	0.160
Hypertension, *n* (%)	58 (60.4)	94 (61.0)	0.922
Diabetes, *n* (%)	14 (14.5)	32 (20.7)	0.219
Hyperlipidemia, *n* (%)	11 (11.4)	21 (13.6)	0.616
Atrial fibrillation, *n* (%)	35 (35.4)	38 (24.6)	0.046
Prior stroke, *n* (%)	11 (11.4)	15 (9.7)	0.665
**Laboratory data**			
Eosinophil, (× 10^9^/l)	0.01 (0–0.02)	0.10 (0.07–0.17)	< 0.001
Neutrophil, (× 10^9^/l)	6.75 (5.25–8.98)	4.55 (3.40–5.62)	< 0.001
ENR × 10^2^	0.15 (0–0.47)	2.45 (1.46–3.75)	< 0.001
Stroke subtype, *n* (%)			0.005
Cardioembolic	54 (56.2)	52 (33.7)	
Atherosclerotic	27 (28.1)	63 (40.9)	
Small vessel/lacunar	6 (6.2)	20 (12.9)	
Cryptogenic/others	9 (9.3)	19 (12.3)	
Onset to needle time (min)	163 (125–200)	150 (121–205)	0.270
Door to needle time (min)	60 (47–85)	58 (44–73)	0.206
Baseline NIHSS scores	11 (7–17)	7 (4–9)	< 0.001
3-month mRS scores	3 (1–6)	1 (0–2)	< 0.001

*ENR, eosinophil-to-neutrophil ratio; NIHSS, National Institute of Health Stroke Scale; mRS, modified Rankin Scale. Among the eligible 266 patients, 16 patients were lost to follow-up and the remaining 250 patients were included in the 3-month prognosis analysis*.

### Lower ENR Level Is Related to a 3-Month Poor Function Outcome

**Figure 3:**
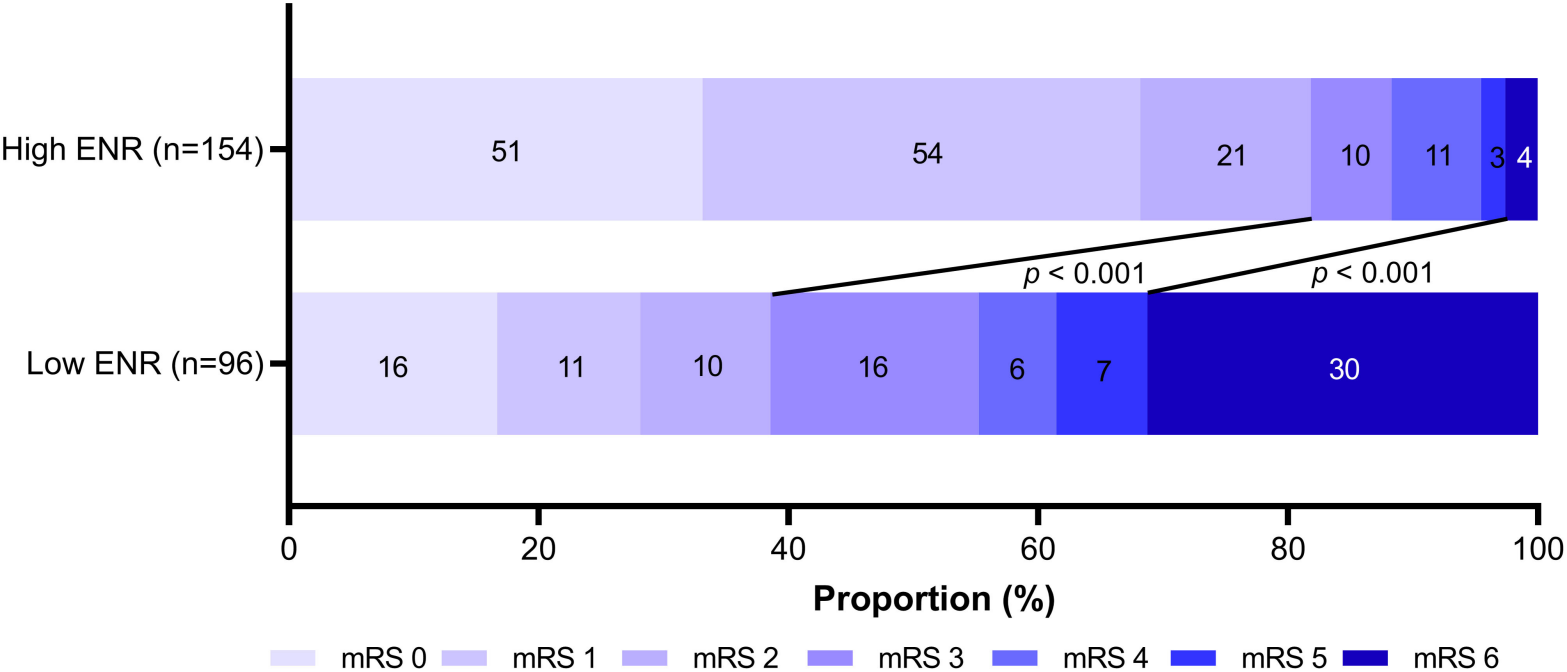
mRS distribution at 3 months for the high-ENR group (ENR × 10^2^ ≥ 0.74) vs. low-ENR group (ENR × 10^2^ < 0.74). mRS, modified Rankin Scale; ENR, eosinophil-to-neutrophil ratio.

**Figure 4:**
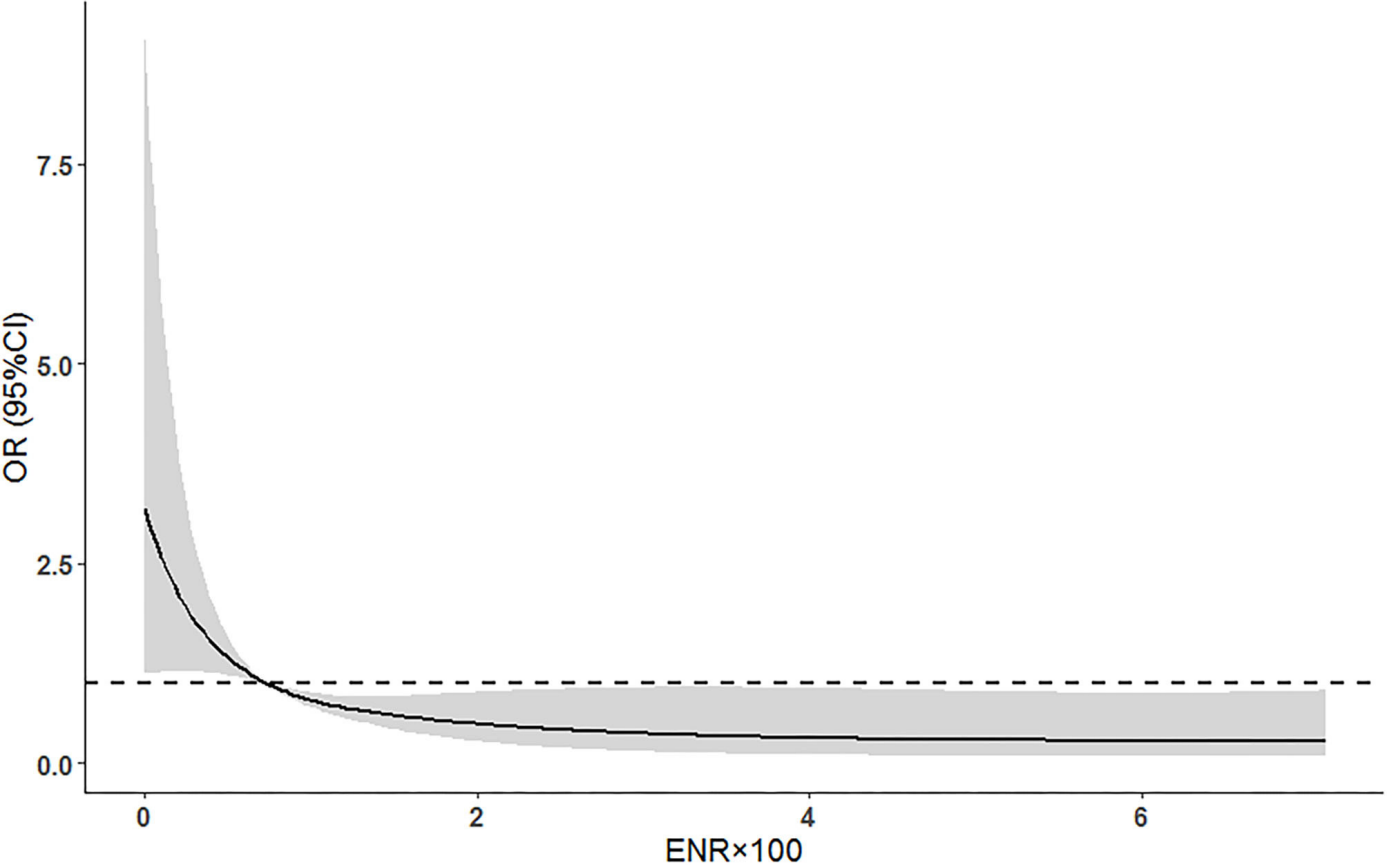
Adjusted association of ENR with 3-month poor outcomes using multiple spline regression analyses with four knots (at the 5th, 35th, 65th, and 95th percentiles). The solid line indicates odds ratio while the shadow indicates 95% CIs. The dashed line is the reference line (odds ratio = 1). The reference of ENR was 0.74. Data were adjusted for age, current smoking, hypertension, atrial fibrillation, prior stroke, baseline NIHSS score, and stroke subtype. ENR, eosinophil-to-neutrophil ratio; NIHSS, National Institute of Health Stroke Scale.

Among 250 AIS patients included in the 3-month prognosis analysis, 87 (34.8%) had poor function outcome. In this cohort, patients in the high-ENR group had a decreased 3-month poor outcome (28 [18.2%] vs. 59 [61.5%], *p* < 0.001) and mortality (4 [2.6%] vs. 30 [31.3%], *p* < 0.001; Figure 3) compared to those in the low-ENR group. After adjusting for potential confounders (age, current smoking, hypertension, atrial fibrillation, prior stroke, baseline NIHSS score, and stroke subtype), multivariate logistic regression showed that high neutrophil and low eosinophil are two independent risk factors for poor 3-month function outcome (Table 3). High ENR (ENR × 10^2^ ≥ 0.74) was independently associated with 3-month function outcome (OR = 0.163, 95% CI 0.076–0.348, *p* < 0.001) and mortality (HR = 0.107, 95% CI 0.030–0.386, *p* = 0.001). Besides, the ENR as a continuous variable was also inversely associated with 3-month poor outcome (per one-point increase of ENR × 10^2^, OR = 0.704, 95% CI 0.560–0.885, *p* = 0.003). In a multivariate logistic regression model with restricted cubic splines, the elevated ENR level was associated with lower odds of 3-month poor outcome (*p* overall association <0.001; Figure 4).

**Table 3:** Univariate and multivariate logistic regression analysis for 3-month poor outcome.

**Variables**	**Univariate analysis**		**Model 1 + eosinophil**		**Model 1 + neutrophil**		**Model 1 + ENR**	
	**OR (95% CI)**	***p*-value**	**OR (95% CI)**	***p*-value**	**OR (95% CI)**	***p*-value**	**OR (95% CI)**	***p*-value**
Age	1.059 (1.033–1.087)	<0.001	1.051 (1.016–1.087)	0.004	1.047 (1.013–1.084)	0.007	1.061 (1.024–1.101)	<0.001
Sex (male)	0.762 (0.446–1.300)	0.318						
Current smoking	0.380 (0.185–0.779)	0.008						
Hypertension	1.586 (0.918–2.739)	0.098						
Diabetes	0.785 (0.394–1.566)	0.492						
Hyperlipidemia	1.545 (0.728–3.280)	0.258						
Atrial fibrillation	1.879 (1.072–3.293)	0.028						
Prior stroke	2.017 (0.895–4.591)	0.090						
Baseline NIHSS score	1.260 (1.180–1.345)	<0.001	1.215 (1.131–1.305)	<0.001	1.197 (1.115–1.285)	<0.001	1.180 (1.096–1.271)	<0.001
**Stroke subtype**								
Cardioembolic	Reference							
Atherosclerotic	0.422 (0.233–0.764)	0.004						
Small vessel/lacunar	0.040 (0.005–0.318)	0.002						
Cryptogenic/others	0.415 (0.168–1.026)	0.057						
Eosinophil (per 0.01 increase)	0.922 (0.884–0.961)	<0.001	0.943 (0.900–0.987)	0.012				
Neutrophil	1.406 (1.237–1.598)	<0.001			1.391 (1.187–1.629)	<0.001		
ENR × 10^2^ (≥0.74)	0.139 (0.078–0.249)	<0.001					0.163 (0.076–0.348)	<0.001

*ENR, eosinophil-to-neutrophil ratio; NIHSS, National Institute of Health Stroke Scale*.

*Model 1 included age, current smoking, hypertension, atrial fibrillation, prior stroke, baseline NIHSS score, and stroke subtype*.

### Secondary Analysis for the Primary Outcome

Sensitivity analyses were employed to test the robustness of our results. The association between ENR and poor 3-month outcome was significant in AIS patients admitted to the hospital during 2016–2017, AIS patients admitted to the hospital during 2018–2019, cardioembolic AIS patients, and non-cardioembolic AIS patients. In addition, these associations were highly robust across the range of decile ENR cutoffs. A higher ENR was associated with significantly better 3-month function outcomes for decile cutoffs from the 20th to 80th percentiles (Table 4). C-statistics, NRI, and IDI were used to verify whether adding ENR to a model containing conventional risk factors could improve the risk stratification of the poor 3-month outcome. Results show that the discriminatory ability of the model for primary outcome significantly improved after adding the ENR (AUC improved by 0.036, *p* = 0.024; NRI 86.71%, *p* < 0.001; IDI 7.92%, *p* < 0.001; Table 5).

**Table 4:** OR (95% CI) of poor 3-month outcomes according to ENR: sensitivity analysis.

	**OR (95% CI)**	***p*-value**
**High ENR vs. low ENR (cutoff = 0.74)**		
Patients from 2016 to 2017	0.277 (0.110–0.699)	0.007
Patients from 2018 to 2019	0.043 (0.008–0.217)	<0.001
Excluded cardioembolic AIS	0.085 (0.025–0.292)	<0.001
Only cardioembolic AIS	0.259 (0.090–0.745)	0.012
**Using different ENR cutoff values**		
ENR top 10% vs. bottom 90%	0.382 (0.098–1.496)	0.167
ENR top 20% vs. bottom 80%	0.326 (0.121–0.877)	0.026
ENR top 30% vs. bottom 40%	0.322 (0.143–0.727)	0.006
ENR top 40% vs. bottom 60%	0.287 (0.137–0.603)	0.001
ENR top 50% vs. bottom 50%	0.241 (0.119–0.488)	<0.001
ENR top 60% vs. bottom 40%	0.163 (0.076–0.348)	<0.001
ENR top 70% vs. bottom 30%	0.249 (0.116–0.531)	<0.001
ENR top 80% vs. bottom 20%	0.200 (0.083–0.479)	<0.001
ENR top 90% vs. bottom 10%	0.451 (0.172–1.183)	0.106

*ENR, eosinophil-to-neutrophil ratio; NIHSS, National Institute of Health Stroke Scale*.

*Adjusted for age, current smoking, hypertension, atrial fibrillation, prior stroke, baseline NIHSS score, and stroke subtypes (Model 1 in Table 3, stroke subtypes were not adjusted in cardioembolic and non-cardioembolic AIS patient groups)*.

**Table 5:** C-statistics and reclassification analyses for ENR to improve the risk stratification of poor 3-month outcome.

	**C-statistics**		**Continuous NRI, %**		**IDI, %**	
	**Estimate (95% CI)**	***p*-value**	**Estimate (95% CI)**	***p-*value**	**Estimate (95% CI)**	***p-*value**
Model 1	0.845 (0.794–0.887)		Reference		Reference	
Model 1 + ENR	0.881 (0.834–0.918)	0.024	86.71 (63.02–110.39)	<0.001	7.92 (4.22–11.61)	<0.001

*The model 1 was included age, current smoking, hypertension, atrial fibrillation, prior stroke, baseline NIHSS score, and stroke subtype*.

*ENR, eosinophil-to-neutrophil ratio; NRI, net reclassification improvement; IDI, integrated discrimination improvement; NIHSS, national institute of health stroke scale*.

### Survival Analysis of ENR Levels and the 1-Year Prognosis

**Figure 5:**
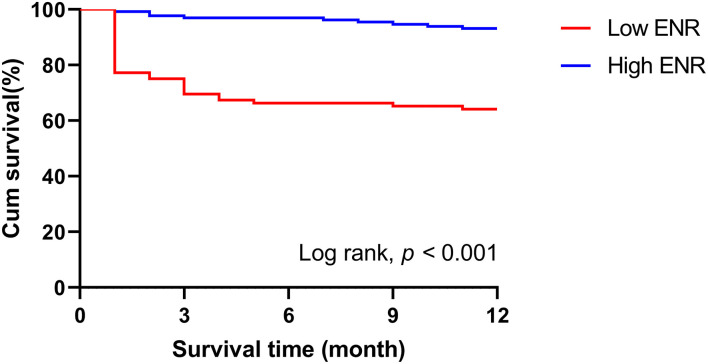
Kaplan–Meier curves comparing the death rate of the two groups over the 1-year follow-up. ENR, eosinophil-to-neutrophil ratio.

At the 1-year follow-up, 43 (16.2%) patients were lost to follow-up and the remaining 223 patients were included in the prognosis analysis. Seventy-seven (34.5%) patients had a poor function outcome and 42 (18.8%) patients had died during the 1-year follow-up. After adjusting for age, current smoking, hypertension, atrial fibrillation, prior stroke, baseline NIHSS score, and stroke subtype, it is interesting that patients with ENR × 10^2^ ≥ 0.74 were more likely to come to a good outcome then those with ENR × 10^2^ < 0.74 (OR = 0.282, 95% CI 0.124–0.639, *p* = 0.002), although no association was found between ENR and 1-year poor outcome when ENR was calculated as a continuous variable. Kaplan–Meier curves and the log-rank test indicated that patients in the high-ENR group had a lower incidence of mortality at the 1-year follow-up (Figure 5). Multivariate Cox regression proportional hazard model analyses were used after adjusting the potential confounders. Patients with a higher ENR were associated with a lower mortality risk (high ENR vs. low ENR: HR = 0.314; 95% CI, 0.135–0.731; *p* = 0.007 and per one-point increase of ENR × 10^2^: HR = 0.586; 95% CI, 0.384–0.872; *p* = 0.008).

## Discussion

A significantly decreased ENR level was observed in the AIS patients compared with the healthy controls. The ROC curve showed that ENR was a fair prognostic biomarker for 3-month poor outcome and had a higher predictive power than either eosinophil or neutrophil count alone. Patients with lower ENR levels were more likely to develop cardioembolic stroke and severe symptoms. In addition, the multivariate adjusted model and restricted cubic splines showed that elevated ENR levels were associated with a lower risk of poor 3-month function outcome. Furthermore, addition of ENR to the conventional model led to the improvement in the model's ability to predict a 3-month poor outcome. Our study also demonstrated that ENR is an independent predictor of 3-month and 1-year mortality in patients with AIS.

ENR is a composite marker of absolute blood eosinophil and neutrophil counts. Neutrophils are most abundant circulating white blood cells and play a vital role during acute inflammatory responses (12). In AIS patients, neutrophils are rapidly recruited into the injury site after stroke onset and release reactive oxygen species (ROS), various proteases, and numerous inflammatory mediators which contribute to tissue damage within the ischemic area (13, 14). A recent study showed that the extracellular traps released by neutrophils are harmful to vascular remodeling after AIS, and an increased extravasation of immune cells and toxic proteins will be observed due to blood–brain barrier (BBB) disruption (15). In addition, the activation kinetics of neutrophils in response to r-tPA should be concerned. Administration of r-tPA can promote *in vitro* and potentially *in vivo* neutrophil degranulation (16). Degranulation products like matrix metalloproteinase-9 (MMP-9) and myeloperoxidase (MPO) are generally considered to be associated with the presence of hemorrhage and poor function outcomes after stroke (17). Maestrini et al. found that higher neutrophil counts and MPO levels were associated with 3-month worse outcomes and higher mortality rates, suggesting that MPO could be a potential therapeutic target (18). In our study, higher neutrophil counts were found in AIS patients compared with healthy controls.

Eosinophils are involved in local immune and inflammatory responses, and treatment targeting eosinophils may help to control a variety of diseases, including atopic diseases such as asthma and allergies, as well as diseases not primarily related to eosinophils, such as autoimmunity and malignancies (19). However, few studies have reported the role of eosinophil in stroke. Eosinophilia has been reported as a prothrombotic condition (20). It is interesting that lower eosinophil counts are associated with severe symptom and poor prognosis of AIS patients (21, 22). The underlying mechanism of eosinophils in stroke is complex, and whether eosinophils are beneficial or harmful depends on the patient's specific background. Enhanced procoagulant activity and impaired anticoagulant properties of the endothelial membrane may contribute to the thrombosis. Eosinophils can release fibroblast growth factor (FGF2), nerve growth factor (NGF), and vascular endothelial growth factor (VEGF), which are involved in vascular remodeling (23). It is worth noting that eosinophil-derived cytotoxic proteins also played an important role in AIS (24). Eosinophil infiltration may be an essential mechanism to explain why eosinophils decreased after stroke. Eosinopenia-producing substances by neutrophils might lead to local margination of eosinophils and thereby cause continued eosinopenia (6). Hence, we may miss the interaction between eosinophil and neutrophil and underestimate the role these cells played in the pathogenesis of AIS if we analyze them separately.

To the best of our knowledge, our study is the first to suggest the association between the ENR level and prognosis of AIS patients treated with intravenous thrombolysis. In regions with different levels of medical resources, a complete blood cell test is widely used. Eosinophils and neutrophils could be obtained and calculated rapidly from a blood sample, which assists clinicians to judge the prognosis of patients at an early stage.

However, several limitations of our study should be acknowledged. First, this study is an observational study and residual confounding still remained. Therefore, the causal relationship between ENR and poor prognosis is unable to establish. Second, the sample size of our study was relatively small; among the 266 patients who met the inclusion criteria, only 250 (94.0%) patients finished the 3-month follow-up and 223 (83.8%) patients finished the 1-year follow-up. Furthermore, subjects of our study were selected from a single hospital so that selection bias may exist in our study.

## Conclusion

Our study shows that a lower ENR is independently associated with 3-month poor outcome and 3-month and 1-year mortality in AIS patients treated by r-tPA intravenous thrombolysis. Monitoring ENR at an early stage might be helpful for risk stratification and making therapeutic decisions.

## Data Availability Statement

The raw data supporting the conclusions of this article will be made available by the authors, without undue reservation.

## Ethics Statement

The studies involving human participants were reviewed and approved by the Ethics Committee of the Third Affiliated Hospital of Wenzhou Medical University. Written informed consent for participation was not required for this study in accordance with the national legislation and the institutional requirements.

## Author Contributions

SZ and GC conceptualized this work and supervised the study. HC, HH, CY, JR, JW, BG, WP, FS, XZ, TZ, JH, and YC acquired the data. HC, HH, CY, and JR performed the statistical analysis and interpreted the data. HC, HH, and CY prepared the manuscript. SZ, GC, HC, HH, CY, JR, JW, BG, WP, FS, XZ, TZ, JH, and YC revised the manuscript. All authors approved the protocol.

## Conflict of Interest

The authors declare that the research was conducted in the absence of any commercial or financial relationships that could be construed as a potential conflict of interest.
